# Molecular Dambusters: What Is Behind Hyperpermeability in Bradykinin-Mediated Angioedema?

**DOI:** 10.1007/s12016-021-08851-8

**Published:** 2021-03-16

**Authors:** Márta L. Debreczeni, Zsuzsanna Németh, Erika Kajdácsi, Henriette Farkas, László Cervenak

**Affiliations:** 1 Research Laboratory, Department of Internal Medicine and Haematology, Semmelweis University, Budapest, Hungary; 2 ELKH-SE Research Group of Immunology and Haematology, Eötvös Loránd Research Network and Semmelweis University, Budapest, Hungary; 3 Hungarian Angioedema Center of Reference and Excellence, Department of Internal Medicine and Haematology, Semmelweis University, Budapest, Hungary

**Keywords:** Endothelial cells, Angioedema, Permeability, Bradykinin, Pathomechanism

## Abstract

In the last few decades, a substantial body of evidence underlined the pivotal role of bradykinin in certain types of angioedema. The formation and breakdown of bradykinin has been studied thoroughly; however, numerous questions remained open regarding the triggering, course, and termination of angioedema attacks. Recently, it became clear that vascular endothelial cells have an integrative role in the regulation of vessel permeability. Apart from bradykinin, a great number of factors of different origin, structure, and mechanism of action are capable of modifying the integrity of vascular endothelium, and thus, may participate in the regulation of angioedema formation. Our aim in this review is to describe the most important permeability factors and the molecular mechanisms how they act on endothelial cells. Based on endothelial cell function, we also attempt to explain some of the challenging findings regarding bradykinin-mediated angioedema, where the function of bradykinin itself cannot account for the pathophysiology. By deciphering the complex scenario of vascular permeability regulation and edema formation, we may gain better scientific tools to be able to predict and treat not only bradykinin-mediated but other types of angioedema as well.

## Introduction

**Fig. 1:**
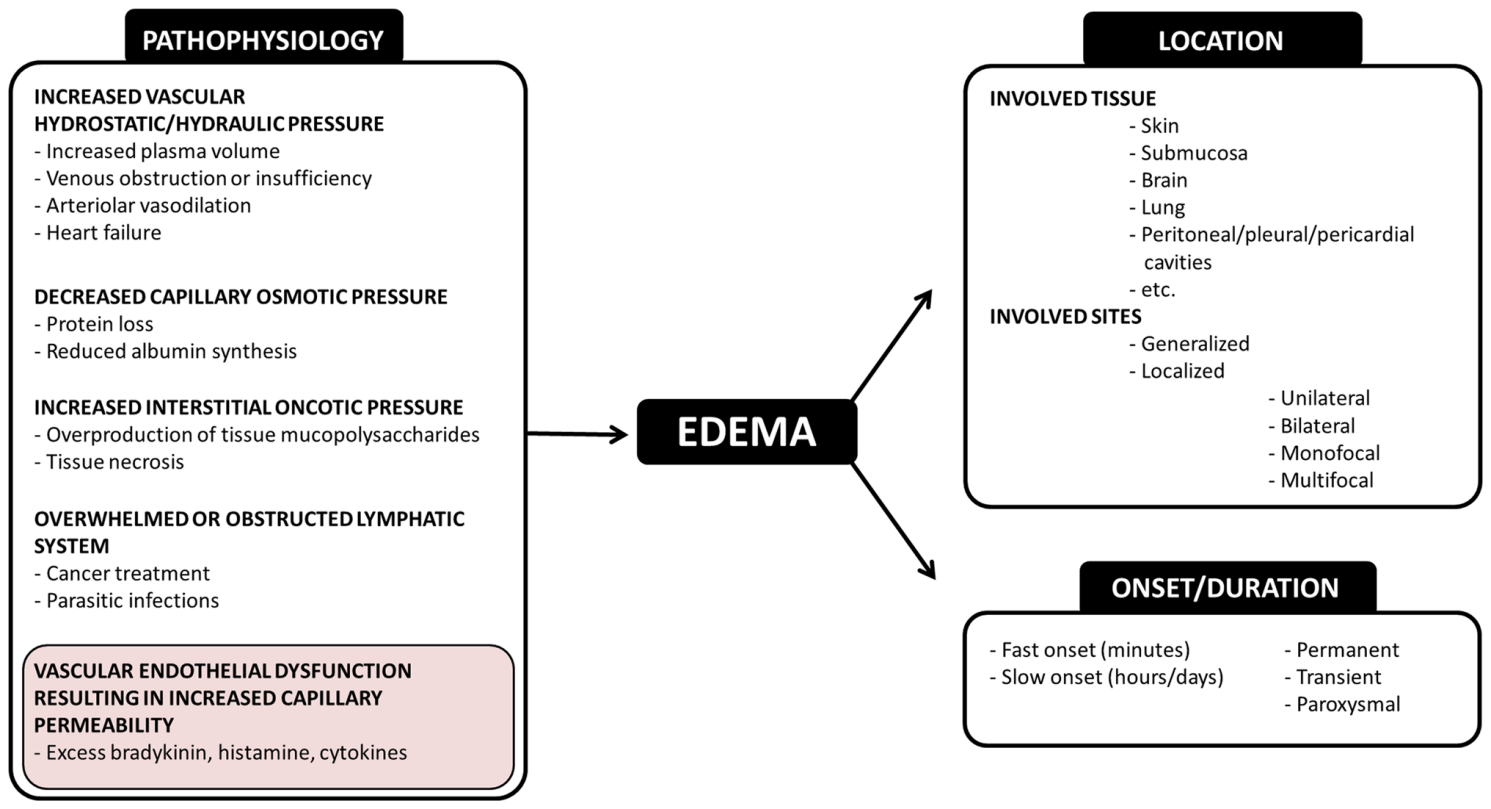
Main features of tissue edema. Pathophysiology, location, and temporal relations are the main features of edema. A few examples are also included under “pathophysiology.” Color shading highlights the main topic of the current review—the vascular endothelium

At the site of microcirculation, convection together with active, directed transcellular transport, and diffusion are responsible for the exchange of metabolites and gases. These processes are majorly controlled by the permeability/barrier (P/B) state of the endothelial cells (ECs). The P/B state depends on the anatomy of capillaries, as adapted to the function of distinct organs. This, however, can also quickly change according to the actual, local requirements. Therefore, increased (as a response) or high (as local tissue characteristics) vascular permeability (IVP/HVP) are not always considered pathological conditions. In other words, IVP/HVP and edema are not synonymous terms. Edema means “a swelling” in ancient Greek (*oidēma*). It occurs when excessive amount of fluid accumulates in the tissues, which may vary in its location, onset, and duration (Fig. 1). According to the Starling equation, edema has two requirements: a driving force of transvascular fluid flux (i.e., a non-zero sum of hydrostatic and osmotic pressure gradient) and the opening of the endothelial barriers (IVP/HVP). A good example is the liver (an HVP organ), where the net water flux is minimal and edema does not normally occur, despite the large intercellular gaps between the sinusoidal ECs. Such an equilibrium exists when the osmotic and hydrostatic pressures are equal between the interstitial and intracapillary space [1, 2]. Moreover, several built-in negative feedback loops exist to avoid the accumulation of fluid in the tissues [3]. Although there are some exceptions, the pathological background of most edemas is well-described. However, through certain vicious circles, multiple factors collaborate in the pathomechanism of almost every edematous disease. This is the case in chronic venous insufficiency, where the venous valvular incompetence (i.e., elevated hydrostatic pressure) induces turbulent blood flow, inadequate perfusion, inflammation, and consequently, increased endothelial permeability, which together lead to limb edema [4].

Although several pathophysiological processes can result in edema formation (Fig. 1), we will focus in this review on types of edema where dysregulation of ECs is the leading pathogenetic factor in the impairment of the P/B state. This is the case in bradykinin (BK)- or histamine-mediated angioedema or in capillary leak syndrome (CLS) [5]. The active participation of ECs in edema can be observed in inflammatory and infectious diseases (i.e., sepsis, and even in COVID-19). In these conditions, regardless of the active or passive role of endothelium, IVP is transiently induced. By eliminating the underlying pathologic factors, the edema also resolves. However, several distinct diseases cause recurrent elevation of EC permeability leading to angioedema attacks without any sign of hypoxia/reoxygenation (H/R) injury, severe inflammation, or injury to the endothelium. Wu et al*.* proposed to name these transient conditions as paroxysmal permeability disorders (PPDs), characterized by the return of the ECs to normal function after resolution of the angioedema attack [6, 7].

Although the term “*angioedema*” should be applied to every edematous disease where the primary cause is the damage or dysregulation of vascular endothelium, it is restrictively reserved to mast cell (MC)-mediated and BK-mediated PPDs. In MC-mediated angioedema, where histamine is the main mediator, a great number of other MC-derived factors and secondary factors produced by other cell types also participate in the pathomechanism. Typically, MC-mediated angioedema has a rather instant onset, beginning within minutes after allergen challenge [8].

As a potent vasoactive oligopeptide, BK has been brought into the focus of research during the exploration of the pathophysiology of angioedema [9]. During an episode of BK-mediated angioedema, BK is overproduced by the uncontrolled activation of the kallikrein-kinin system (KKS) due to C1-inhibitor (C1-INH) deficiency [10], or is elevated due to reduced breakdown caused by angiotensin converting enzyme inhibitors (ACEIs) [11, 12].

The picture of pathophysiology of angioedema is more complicated than we thought earlier. Research in the recent years has shown that BK also has a role in certain types of angioedemas that were not classified as BK-mediated before. For instance, heparin-initiated BK production plays a fundamental role in MC-mediated diseases [13]. It was recently proposed that BK also has a critical role in the lung inflammation of COVID-19. Consequently, targeted therapies originally developed for the management of BK-mediated angioedemas might also be effective for the treatment of COVID-19 [14].

BK-mediated angioedema involves a variety of plasma enzyme cascades (KKS, coagulation, fibrinolysis, and complement) [15, 16], vascular endothelium [7, 17, 18], lymphatic endothelium [19], and cells of the immune system (e.g., neutrophil granulocytes) [20]. Various signaling pathways (such as ANGPT1-Tie2 signaling in HAE patients with *ANGPT1* mutation) [21], hormonal effects [22], the autonomic nervous system [23], and other mechanisms yet to be discovered [24] may also contribute to the pathogenesis.

Maintaining the hemodynamic balance of an organism—including the P/B state of the endothelium—is a complex process, which requires the precise cooperation of many organ systems. The pathophysiology of ECs in C1-INH deficiency provides a good example for such complexity. In this review, we aim to provide an insight into the molecular mechanisms of permeability-increasing and permeability-decreasing agents. Furthermore, we expose some of the current controversies in angioedema from the perspective of ECs, and finally, attempt to outline an alternative, complex hypothesis on the role of ECs in the pathogenesis of BK-mediated angioedema.

## Molecular Mechanisms Behind Regulation of Permeability

When investigating the pathomechanism of edematous conditions, it is of key importance to reveal the P/B state of ECs and identify the relevant permeability modulating agents. To accomplish this, one needs to understand how permeability is regulated at the cellular/molecular level. At physiologic conditions, transcellular transport is controlled by chemical potential-driven diffusion and active vesicle transport, while paracellular transport is controlled by opening gaps between ECs. Transcellular and paracellular transport contribute to the exchange of materials between the blood and the tissues. The proportion of these transport mechanisms are substantially organ dependent. For example, in the brain, transcellular transport is almost exclusive, whereas in sinusoid endothelia (in liver, bone marrow, and spleen) paracellular transport is the dominant mechanism.

**Fig. 2:**
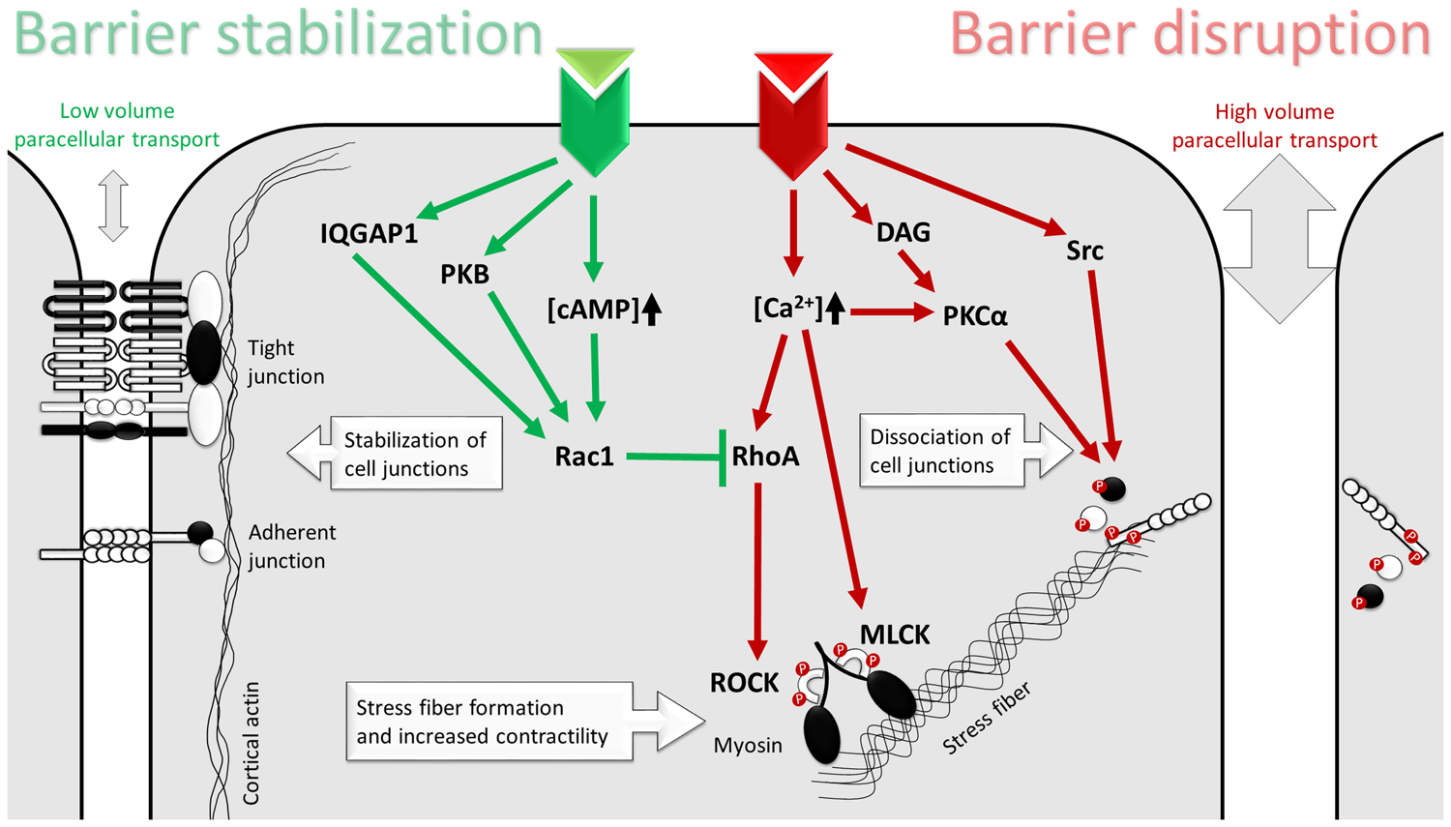
Common intercellular signaling events of endothelial permeability regulation. The most important signaling events leading to barrier protection (tight junction and adherent junction stabilization, formation of cortical actin network) are emphasized by green color, whereas those leading to barrier disruption and hyperpermeability (phosphorylation and dissociation of cell junction components, phosphorylation of myosin light chain and formation of actin stress fibers) by red color

This review will focus on the mechanism of paracellular transport, since it is the predominant route of fluid transport during pathological conditions. To function as a barrier, ECs must have well-organized tight junctions (TJs) involving several adhesion molecules, such as occludin, junctional adhesion molecules (JAMs), claudins, angulins, and tricellulin*.* Through intracellular adaptor proteins from the *zonula occludens* protein family (ZOs), their intracellular domains are connected to actin cytoskeleton. TJs are physically supported by belt-like adherent junctions (AJs) and patchy desmosomes (DSs). Vascular endothelial cadherin (VE-cadherin), the predominant homotypic adhesion molecule of AJs in ECs, is anchored to actin fibers and behaves as a force transducer between ECs [25]. DSs and hemidesmosomes (HDs) are also important structures participating in cell-cell and cell-basal membrane connections [26]. Besides TJs (as complete sealers even for water molecules) and AJs (which retain blood cells and macromolecules with the largest size), *glycocalyx* also contributes to the barrier function by repelling negatively charged molecules, even where TJs are not intact or fully closed [27]. A well-structured cortical actin cytoskeleton is also required for the normal barrier function. Both the members of the cellular adhesion complexes described above and the acto-myosin network are regulated by several mechanisms including phosphorylation/dephosphorylation, internalization of receptors, and adaptor assembly/disassembly controlled by Ca-ions. Normally, these effects are completely reversible in order to effectively restore the original tissue functions. Agonist-induced disruption of the endothelial barrier occurs through the rearrangement of actin cytoskeleton, accompanied by the activation of the contractile apparatus and the disassembly of cellular junctions (especially VE-cadherin containing AJs). As a result, paracellular gaps form, causing endothelial hyperpermeability. The above mechanism is summarized in Fig. 2.

After receptor activation, intracellular Ca^2+^ level rises, which in turn activates myosin light chain kinase (MLCK), RhoA/Rho kinase (ROCK), and protein kinase C (PKC) signaling. These pathways lead to the phosphorylation and activation of myosin light chains (MLC), actin stress fiber formation, increased contractility, and phosphorylation (and thereby dissociation) of intracellular AJ components [28]. This primary process of hyperpermeability takes place rather quickly, within minutes. Then why do some types of edema caused by hyperpermeability develop much slower? This can be the case if agonist receptors and other factors of permeability signaling are induced transcriptionally or if triggering factors act via other cell types, which in turn locally produce hyperpermeability agonists.

It is also important to comprehend how normal barrier function is maintained. The signaling of barrier protective mediators converge predominantly at the level of Rac1, a small GTPase with a crucial role in controlling RhoA activity and the cytoskeletal rearrangement. A well-known pathway leading to Rac1 activation is cyclic adenosine monophosphate (cAMP) signaling. cAMP interferes with RhoA as a result of a very intricate signaling network: either through activation of the PKA/Rac1 axis or Epac signaling (the latter also leads to the stabilization of microtubules) [29]. The signaling of other barrier enhancing mediators can promote Rac1 activation through protein kinase B (PKB/Akt) or Ras GTPase-activating-like protein (IQGAP1) activation, without increasing cAMP production. The importance of the above mentioned barrier stabilizing mechanisms is well illustrated during inflammation or sepsis, where an impaired cAMP/Rac1 axis contributes to the barrier disrupting effects of hyperpermeability mediators [30].

Taken together, theoretically, every mediator influencing the expression or the function of (1) permeability inducing or decreasing factors, (2) their receptors, (3) Ca^2+^ channels, (4) compounds of the Rho/ROCK/MLCK/Rac1 system, (5) cAMP metabolism, or (6) adaptors or adhesion molecules of TJs, AJs, DSs, and HDs can potentially modify endothelial permeability.

## Permeability Modifying Mediators

Molecules known to affect endothelial permeability represent great diversity in their mechanisms of action. For the purpose of this review, we arbitrarily defined permeability modifying factors as “C1-INH sensitive” or “C1-INH non-sensitive.” C1-INH sensitive factors interact with C1-INH (i.e., they can be inhibited by C1-INH, can cleave C1-INH or their metabolism is influenced by C1-INH). The highly effective therapeutic C1-INH concentrates may also act at the level of these factors.

C1-INH non-sensitive factors (i.e., those without any obvious connection with C1-INH) may also attract attention, since they can modify the frequency, severity, localization, and resolution of angioedema. We present permeability modifying mediators in groups based on their structure, receptor usage or origin (Table 1).

**Table 1:** Permeability modifying factors. Upward arrows indicate increased permeability. Downward arrows indicate decreased permeability/protected barrier function. Some of the factors may have both effects on permeability shown by up and down arrows. Interaction with C1-INH indicates if the factor is inhibited by C1-INH, cleaves C1-INH, or its metabolism is influenced by C1-INH

Name	Acronym	Permeability	Interaction with C1-INH
Vasoactive peptides			
Adrenomedullin	ADM	↓	
Angiotensin II	ATII	↓/↑	
Arginine-vasopressin	AVP	↓	
Bradykinin	BK	↑	✓
Calcitonin gene-related peptide	CGRP	↑	
desArg-bradykinin	desArg-BK	↑	
desArg-kallidin		↑	
Endothelin-1	ET-1	↓/↑	
Intermedin	ADM2	↓	
Kallidin		↑	
Natriuretic peptide A	ANP	↓	
Natriuretic peptide B	BNP	↓	
Natriuretic peptide C	CNP	↓/↑	
Neurokinin A	NKA	↑	
Neurokinin B	NKB	↑	
Relaxin		↓	
Substance P	SP	↑	
Protease activated receptor agonists			
C4a		↑	✓
Mannan-binding lectin-associated serine protease 1	MASP-1	↑	✓
Mannan-binding lectin-associated serine protease 2	MASP-2	↑	✓
Plasma kallikrein	PKa	↑	✓
Plasmin		↑	✓
Thrombin		↑	✓
Zonulin		↑	
Amino acid derivatives			
Adrenaline		↓/↑	
Noradrenaline		↓/↑	
Histamine	HA	↑	
Serotonin	5-HT	↑	
Cytokines and growth factors			
Angiopoetin-1	ANG-1	↓	
Angiopoetin-2	ANG-2	↓/↑	
Basic fibroblast growth factor	bFGF	↓	
Epidermal growth factor	EGF	↓	
Interferon $\gamma$	IFN$\gamma$	↑	
Interleukin 1 $\alpha$, $\beta$	IL-1$\alpha$, $\beta$	↑	
Interleukin 1 receptor antagonist	IL1-Ra	↓	
Interleukin 10	IL-10	↓	
Interleukin 18	IL-18	↓/↑	
Transforming growth factor $\beta$	TGF$\beta$	↓/↑	
Tumor necrosis factor $\alpha$	TNF$\alpha$	↑	
Vascular endothelial growth factor A	VEGF-A	↑	
Lipid mediators			
Ceramide		↑	
Platelet activating factor	PAF	↑	
Prostacyclin	PGI2	↓	
Epidermal growth factor	EGF	↓	
Sphingosine-1-phosphate	S1P	↓	
Thromboxane A2	TXA2	↑	
Complement C5a and C1r			
C1r		↑	
C5a		↑	
Purinergic mediators			
Adenosin		↓/↑	
Adenosin triphosphate	ATP	↓/↑	
Steroid compounds			
Estorgens		↓/↑	
Hydrocortisone		↓	
Free radicals, gasotransmitters and pH			
Carbon-monoxide	CO	↓	
Hydrogen peroxide	H_2_O_2_	↓	
Hydrogen sulfide	H_2_S	↓	
Nitrogen monoxid	NO	↓/↑	
pH changes		↑	
Superoxid anion	O_2_^-^	↑	
Microbial compounds			
Cholera toxin (from *Vibrio cholerae*)		↓	
* Clostridium botulinum* toxin C		↑	
* Clostridium difficale* toxin A and B		↑	
Cytochalasin D		↑	
Enterovirus E71 capsid protein VP1		↑	
Lipopolysaccharide	LPS	↑	
Pertussis toxin (from *Bordetella pertussis*)		↑	
SARS-CoV2 spike protein		↑	
Zonula occludens toxin (from Vibrio cholerae)	Zot	↑	

### Vasoactive Peptides and Peptidases

The group of vasoactive peptides comprise small oligopeptides that are usually produced as pro-peptides. They gain activity after cleavage of the pro-peptide, remain active for a relatively short period, bind to cell surface receptors, and can be inactivated by soluble or cell surface peptidases.

#### BK and Kallidin

BK is a 9-amino acid-long oligopeptide, cleaved by plasma kallikrein (PKa) from high molecular weight kininogen (HK) mostly on the surface of ECs. Its predominant role in angioedemas have previously been discussed thoroughly [9, 31–33]. The plasma level of BK was found to be elevated during angioedema attacks [32]. BK represents a C1-INH-sensitive permeability factor, since its production is inhibited by C1-INH. Moreover, complement MASP-1, another serine protease that can be inhibited by C1-INH, is also able to cleave HK and form BK, although with less efficiency than PKa [34]. Kallidin (or Lys-BK) is a 10-amino acid-long oligopeptide, cleaved by tissue kallikrein 1 (KLK1) from low molecular weight kininogen. In contrast to BK, kallidin is C1-INH non-sensitive, as KLK1 is not inhibited by C1-INH [35]. Both BK and kallidin act on BK receptor B2 (BKRB2), and, with a lower affinity, on BKRB1 (both expressed on ECs). The first breakdown products of BK and kallidin, desArg-BK, and desArg-kallidin, respectively, are also regarded as permeability-increasing factors, acting solely on BKRB1. Both BK receptors belong to the G-protein coupled receptor (GPCR) family and induce Ca-signaling, PLA2/C/D, PKCδ, and NFκB activation [36]. They are regulated by GPCR kinase 2 (GRK2), which was recently found to be an important determinant of attack severity in C1-INH-HAE patients [37].

The first step of BK and kallidin breakdown is catalyzed by carboxypeptidase N, which cleaves BK and kallidin into their still vasoactive desArg forms, whereas other peptidases carry on further cleavage, resulting in completely inactive peptide fragments. This metalloproteinase group comprises, amongst others, carboxypeptidase M, neutral endopeptidase, angiotensin-converting enzymes (ACE, ACE2), and dipeptidyl-peptidase (DPP-IV). The latter peptidases can be blocked by ACE inhibitors (ACEI) and SARS-CoV2 or DPP-IV inhibitors (gliptins), respectively, which may all cause drug-induced angioedema by the inhibition of BK/kallidin breakdown [38–40]. Interestingly, BK is much more active as a permeability-increasing factor in vivo than in vitro [41]. A plausible explanation of this discrepancy is that BK is an extremely potent stimulator of the sensory nerves [42], which, in response to BK, produce several permeability increasing or potentiating factors [43, 44].

#### Angiotensin II

Angiotensin II, as the most active component of the renin-angiotensin system, is a very potent blood-pressure elevating vasoactive peptide and also influences permeability. It increased blood–brain barrier (BBB) permeability in murine models via its AT1 receptor, without having direct effects on TJ components or actin cytoskeleton [45]; however, it involves reactive oxygen species [46]. Angiotensin II antagonized the increased microvascular permeability induced by ATP in a rat model [47]. It is worth noting that the effects of angiotensin II on endothelial permeability are, at least partially, mediated by vascular endothelial growth factor (VEGF) [48, 49]. Although angiotensin II can influence vascular permeability, its converting enzymes (ACE and ACE2) are more important permeability regulators as they take part in the breakdown of BK.

#### ET-1

Endothelin-1 (ET-1) is a 21-amino acid peptide, synthesized mostly in ECs. Besides being the most potent vasopressor agent yet identified acting on vascular smooth muscle cells, ET-1 also has receptors on ECs. ET-1 was shown to decrease ATP- or BK-induced permeability as well as hydraulic permeability [50]; however, in other models, it increased or had no effect on vascular permeability in vivo [51–53]. This can partially be explained by the secondary effects of ET-1 as a strong inducer of both permeability-increasing thromboxane A2 (TXA2) and permeability decreasing adrenomedullin (ADM) [51, 54]. It is yet unclear whether the elevated level of ET-1 during C1-INH-HAE attacks [55] indicates a pathogenetic or a moderating role for ET-1 in edema formation.

#### AVP

Arginine-vasopressin (AVP) is an antidiuretic vasoactive nonapeptide of neuronal, cardiac, and adrenal gland origin. However, it also has a barrier stabilizing role, which may result from similar receptor signaling as its antidiuretic effect. AVP alone had no effect on the permeability of the BBB in a rat model [53], but in hamsters, it potently inhibited the permeability-increasing effects of histamine [56]. We previously found that AVP concentration was elevated during attacks in C1-INH-HAE patients, which also suggests its regulatory role in permeability [55].

#### NP Family

Natriuretic peptide (NP) family consists of three structurally similar peptides: the 28-amino acid ANP, the 32-amino acid BNP, and the 22-amino acid CNP. ANP and BNP are predominantly produced by the atrial muscle cells, whereas CNP is synthesized mainly by the vascular ECs. ANP has a well-described permeability decreasing effect. It suppresses both the basal permeability [57], and permeability induced by thrombin [58], tumor necrosis factor alpha (TNFα) [59], oxidants [60], VEGF [61], and histamine [62]. BNP has similar effects to those of ANP, it could also block thrombin-induced permeability [63]; however, the role of CNP is more controversial. Compared to ANP and BNP, CNP had no effect on permeability [63]; however, in another study, it increased BBB permeability by reducing ZO-1 expression [64]. ANP acts predominantly by inducing Rac1/PAK1 activation, stabilization of microtubules, and blocking RhoA pathway [65]. We found that ANP levels were lower during attack-free periods of C1-INH-HAE patients than in controls, suggesting that it is indeed a hyperpermeability factor [66].

#### ADM and the CGRP Family

Adrenomedullin (ADM) is a 52-amino acid peptide of the calcitonin gene-related-peptide (CGRP) family produced by a wide range of tissues including ECs themselves. ADM induces vasodilation, natriuresis, and cell growth. Moreover, it enhances the barrier function of ECs by cAMP induction, adhesion, and adaptor protein recruitment into the TJs and AJs, and Rap1-dependent cytoskeleton stabilization. Using several in vitro as well as in vivo models, ADM effectively decreased both basal and pre-induced permeability [67–70]. We found that ADM concentration was higher during attacks of C1-INH-HAE, than in symptom-free periods [55]. Intermedin (ADM2), another member of the CGRP family, also has barrier protecting activities via cAMP/PKA and cAMP/Epac signaling pathways [71]. Although CGRP, the denominator of the family, released mainly from sensory neurons, has less pronounced direct effects on vascular permeability. Early studies demonstrate a synergistic permeability-increasing role for CGRP [43, 72].

#### SP and NKs

Substance P (SP), NKA, and NKB belong to the family of tachykinins. These three closely related small peptides (11, 10, and 10 amino acids, respectively) mainly produced by sensory neurons are known as permeability-increasing factors.

Of the GPCR family neurokinin (NK) receptors, NK_1_ mediates the most pronounced permeability-increasing effect on ECs [73]. SP possesses the highest affinity to NK_1_ and is the strongest permeability-increasing factor in the tachykinin family. Although most studies concentrate on the SP/BBB interaction, SP acts on lung and skin vasculatures as well [44]. The neurogenic inflammation mediated by SP also has indirect effects on vascular permeability, as it is a potent histamine releasing factor [74]. The tachykinin family members are also prone to cleavage by ACE, ACE2, neutral endopeptidase and other cell surface peptidases.

#### Relaxin

Relaxin is a 52-amino acid polypeptide, a member of the insulin family, produced predominantly by the reproductive systems and the heart. Although it can induce VEGF expression [75, 76] leading to a late-onset increase in permeability, in short-term, relaxin is a potent barrier protecting factor. It prevents the permeability-increasing effects of H/R injury, hydrogen peroxide (H_2_O_2_) and cytokines [77].

### PAR Agonists

Most BK-mediated angioedemas are closely related to plasma enzyme cascade systems involving more than 20 enzymes. The majority of these enzymes belong to the serine protease family and many of them are inhibited by C1-INH. Therefore, except when specifically the BK breakdown is impaired, disturbances of these cascade systems are observed in BK-mediated angioedemas [78, 79].

The participating enzymes, as well as several other plasma and tissue enzymes, act on ECs via protease activated receptors (PARs) and in that way they can cause increased vascular permeability (Table 2). PARs, belonging to the GPCR family, have a unique activation mechanism [80]. They are activated by a proteolytic cleavage on their N-terminal domain and the newly acquired N-terminal sequence becomes a tethered ligand, which leans back to the extracellular part of the 7TM domain and activates the receptor. PARs (PAR1-4) are expressed ubiquitously in the human body, but predominantly on vascular, epithelial, intestinal, and immune cells, as well as the nervous system. PARs can be cleaved at canonical or non-canonical cleavage sites, resulting in diverse signaling and cellular responses. The non-canonical cleavage causes the so-called biased signaling (incomplete or different signaling compared to the ones that have been described originally) [80], which has been observed in the case of all PARs [81–86]. In the following discussion, we describe the permeability modulating effects of a few important serine proteases of the PAR agonist group, as well as the non-enzymatic PAR agonist C4a, and how PAR-cleaving enzymes and other agonists act on vascular permeability.

**Table 2:** Protease activated receptor agonists

	Relations to C1-INH	Protease group	Receptor			
			PAR1	PAR2	PAR3	PAR4
Activating proteases	Inhibited by C1-INH	Serine proteases	Kallikrein-5, MASP-1, plasma kallikrein, plasmin, thrombin	Kallikrein-5, MASP-1, plasma kallikrein, plasmin	Plasmin, thrombin	MASP-1, plasmin, thrombin
	Not inhibited by C1-INH	Serine proteases	Cathepsin G, Factor VIIa, factor Xa, granzyme A, B, K, kallikrein 4, 6, 14, proteinase 3	Acrosin, cathepsin G, factor VIIa, factor Xa, granzyme A kallikrein 4, 6, 14, mast cell tryptase, matriptase, proteinase 3, testisin, tryptase	Factor Xa	Factor VIIa, factor Xa, cathepsin G, kallikrein 14
		Cysteine proteases	Calpain-1, gingipain	Calpain-1, cathepsin S, gingipain		Gingipain
		Metalloproteases	MMP-1, MMP-2, MMP-3, MMP-8, MMP-9, MMP-12, MMP-13, MMP-14			
	Inactivates C1-INH	Serine proteases	Neutrophil elastase, trypsin	Elastase, neutrophil elastase, trypsin	Trypsin	Trypsin
Other agonists	C1-INH sensitive		C4a			C4a
	Not inhibited by C1-INH			Zonula-occludens toxin, zonulin		

#### Thrombin

Thrombin is a multifunctional serine protease being cleaved and activated from prothrombin in the blood. It participates in blood coagulation by forming fibrin from fibrinogen, and by converting coagulation factors FXI, FVIII, FV, and FXIII to their active forms. It also has major pro-inflammatory effects on ECs [87]. Thrombin cleaves PAR1 and PAR4, but it cannot activate PAR2 [88], while its PAR3 activation capability is still controversial. Thrombin directly increases endothelial permeability [89] via PAR-1 activation [90, 91] by disrupting the VE-cadherin/catenin complexes [92] in the AJs.

#### MASP-1

Mannan-binding lectin-associated serine protease 1 (MASP-1) is best known as being a key component in the activation of complement lectin pathway [93]. Our group showed that, similarly to thrombin, it directly activates ECs via PARs. The difference is their enzymatic activities and receptor preferences, i.e., MASP-1 can cleave PAR2 in addition to PAR1 and PAR4 [94]. The receptor activation manifests in pro-inflammatory activation of ECs [87, 94–96] and increased endothelial permeability by rearranging the actin-cytoskeleton, disrupting TJ/AJ components and altering transcription of several permeability related proteins [97].

#### MASP-2

Mannan-binding lectin-associated serine protease 2 (MASP-2) is activated by MASP-1, and by cleaving C2 and C4 it has a predominant role in complement lectin pathway activation. Using a human umbilical vein EC (HUVEC) model, we showed that MASP-2 is also a potent permeability-increasing enzyme. Although both the mechanism and the kinetics are very similar to those of thrombin and MASP-1 [98], the involvement of PARs in its activity is yet to be proven. We found a significant increase in MASP-2 levels in the 1/3 of patients during angioedema attacks [99], which supports the notion that MASP-2 may contribute to edema formation.

#### PKa

Plasma kallikrein (PKa) is formed from prekallikrein (PK) by FXIIa. It releases BK from HK, cleaves FXII and plasminogen, and is able to activate ECs via PAR1 and PAR2 [100, 101]. Increased leukocyte trafficking and enhanced ICAM-1 and VCAM-1 expression was demonstrated in the presence of PKa [101], which may modulate permeability indirectly through pro-inflammatory cytokines. PKa can cleave endothelial VE-cadherin in AJs [102]; moreover, as we have shown, it can activate ECs (possibly via PAR cleavage) and thereby directly increases endothelial permeability [98].

#### Plasmin

Plasmin is released from the inactive precursor plasminogen and after binding to clots or cell surfaces, can be activated by a variety of enzymes (e.g., tPA, uPA, PKa, FXII) [103]. Plasmin is best known as a fibrinolytic enzyme, but it can also activate several types of collagenases and cleave HK to BK [104]; furthermore, it cleaves all four types of PARs [88]. Although various permeability-increasing effects of plasmin were demonstrated (e.g., proteolytic damage of ECs, activation of matrix metalloproteinase MMP-3/9), the exact molecular mechanism is still controversial [105, 106]. Tranexamic acid, an antifibrinolytic drug, inhibits the formation of plasmin and has long been used in the prophylaxis of BK-mediated angioedema. Interestingly, it is also capable of protecting EC glycocalyx integrity independent of plasmin action, and thus protects barrier function [107].

#### Complement Fragment C4a

C4a, a complement activation product formed by C1s or MASP-2 (both are C1-INH sensitive serine proteases), turned out to increase endothelial permeability as an untethered agonist for PAR1 and PAR4 [108].

#### Signaling of PAR Agonists

PARs can signal through G-protein subunits Gβγ and almost all the Gα-s, which are major regulators of endothelial permeability. In general, the activation of the Gα_q_ and Gα_o_ subunits activates PLC-β, the Gα_i_ subunit is responsible for adenylate cyclase inhibition, while Gα_12/13_ activation induces GTPases from the Rho signaling pathway and this way regulates actin cytoskeleton.

In ECs, PAR1 is coupled with Gα_q_ [109, 110], Gα_i_ [111], and Gα_12/13_ [110, 112], while PAR2 is associated with Gα_q_ [113] subunits. We and others showed that PAR1 agonists are very potent permeability-increasing factors of human ECs in vitro [97, 114] as well as ex vivo in mice [90] and in vivo rats [115]. In human ECs, PAR1 agonists induce strong RhoA activation [114], Ca^2+^ influx [97], formation of actin stress fibers [114], and ring-like actin filaments [116], as well as inhibition of Rac [117]. PAR2 agonists also induce RhoA activation [114], Ca^2+^ influx [97], and actin stress fiber formation [114]; however, they can also stabilize the barrier function of human ECs via Rac activation [117]. Interestingly, although PAR2 agonists induce edema when injected into rat paws [118], this effect is obtained indirectly, by the release of vasoactive peptides (i.e., SP and CGRP), which originate from the sensory nerves activated by the PAR2 agonist [119]. This effect of PAR2 activation nicely demonstrates that hyperpermeability can be effectively induced indirectly, by utilizing other cell types (just as in the case of BK). PAR4 agonists induce actin stress fiber formation [116] and a weak Ca^2+^ influx [97] in human ECs. Jong-Sup et. al showed that PAR4 also plays a direct role in increasing endothelial permeability. When they blocked PAR1 with an antagonist peptide, thrombin could still induce permeability change in the endothelial layer [120], and we showed that PAR4 agonist directly increased endothelial permeability in HUVECs [97]. The role of PAR3 in the regulation of permeability is still unexplored. The role of PARs in edema formation is further complicated by simultaneous signaling and inhibition by protease catalyzed disarming [121, 122].

### Amino Acid Derivatives

#### Histamine

Histamine is a well-known permeability-increasing agonist, synthesized by the decarboxylation of the amino acid histidine. Being produced and stored in mast cells and basophil granulocytes, it is the main mediator of acute allergic reaction. Its permeability-increasing effect causes edema and anaphylactic shock [123, 124]. Histamine has four receptors on ECs (H_1_R to H_4_R), and although all of them are GPCRs, they have opposing effects on endothelial permeability. H_1_R is responsible for the majority of the histamine-induced endothelial hyperpermeability. H_1_R is coupled to Gα_q_ and its activation leads to intracellular Ca^2+^ mobilization, and activates MLCK, RhoA/ROCK, and p38 MAP kinase signaling, resulting in MLC phosphorylation, actin stress fiber formation, and cell contraction. This causes a rapid and fast-expiring increase in endothelial permeability (maximal response in 3 min) [125, 126]. The Gα_s_-coupled H_2_R has the opposite effect, it stabilizes the endothelial barrier as it stimulates the production of cAMP [127]. The mechanism of action of H_3_R and H_4_R is less understood, but these receptors are thought to contribute to the permeability-increasing effect of histamine in certain tissues, such as the skin [125].

#### Serotonin

Serotonin (or 5-hydroxytryptamine, 5-HT) is produced from tryptophan by enterochromaffin cells and MCs. It is released into the circulation, from where platelets take it up and store it in dense granules. Upon inflammation, platelets release serotonin back into the circulation, which leads to increased endothelial permeability, as demonstrated in endotoxemia [128, 129].

#### Catecholamines

Catecholamines (i.e., dopamine, adrenaline, noradrenaline) are the derivatives of phenylalanine/tyrosine and comprise several neurotransmitters and hormones. The permeability modulation by catecholamines is rather complex and sometimes controversial. Although the effect of adrenaline on β-adrenergic receptors induce cAMP synthesis, leading to decreased permeability [130], it may also induce α-adrenergic receptors and the breakdown of EC glycocalyx, both resulting in diminished barrier function [107, 131]. Noradrenaline may also act as a permeability-increasing or decreasing factor depending on the actual state of the ECs and their surroundings [132, 133]. Interestingly, no effects of catecholamines on lung microvascular permeability was found in ex vivo experiments [134]. Nevertheless, the direct effects of catecholamines may be exceeded by their secondary impact on the production and accessibility of other permeability modulating factors, such as ET-1 [135] and VEGF [136].

### Cytokines and Growth Factors

Several cytokines affect endothelial/epithelial barrier function to either increase or decrease monolayer permeability. It is no surprise that pro-inflammatory cytokines, such as tumor necrosis factor-α (TNFα), interferon-γ (IFNγ), the majority of the interleukin-1 (IL-1) family, IL-4, IL-6, IL-8, and IL-13 act mostly as permeability-increasing agonists, while anti-inflammatory cytokines (IL-1Ra, IL-10) tend to protect the endothelial barrier. Although cytokines can modify vascular permeability directly by initiating intracellular signaling pathways in ECs, they also have pleiotropic secondary permeability-regulating effects via activation or suppression of leukocytes and other non-endothelial cell types. The vascular effects of cytokines are observed in the case of capillary-leak syndromes, referred to as “cytokine-storm,” but rarely connected to BK-mediated angioedema. Growth factors (GFs) regulate the proliferation and differentiation of various cell types and will be discussed here together with the cytokines because their signaling events and effects have many common characteristics.

#### TNFα

Tumor necrosis factor alpha (TNFα) is a potent pro-inflammatory cytokine that can increase endothelial permeability via the receptor TNFR1 or the combined signaling of TNFR1/TNFR2, although the underlying mechanisms are still quite elusive [41, 137]. The delayed but prolonged hyperpermeability effect of TNFα (8–24 h) was suggested to be caused by the removal/redistribution of TJ proteins without early RhoA activation and MLC phosphorylation [138], but in a more recent study, ROCK and MLCK inhibitors prevented the TNFα-induced hyperpermeability in vitro [139]. Moreover, TNFα stimulation upregulates the expression of TRPC1, the predominant isoform of store-operated cation channel in ECs, which results in an augmented Ca^2+^ influx, and, consequently, an enhanced permeability increase in response to other GPCR-utilizing agonists [140].

#### IFNγ

Interferon gamma (IFNγ) is predominantly produced by T_H_1, T_C_1, and NK cells during viral infections. The permeability-increasing effect of IFNγ is long-known [141]. There is one known receptor for IFNγ, IFNGR, that is comprised of two ligand-binding IFNGR1 and two associated signal transducing IFNGR2 chains [142]. Results of in vitro experiments on endothelial/epithelial monolayers showed that IFNγ induces the activation of the p38 MAPK and the RhoA/ROCK axis, resulting in MLC phosphorylation, acto-myosin contraction, cell-shape changes, and internalization of TJ-associated adhesion molecules [143, 144]. Nevertheless, IFNγ cannot induce a rapid permeability change but has a remote effect on endothelial permeability, which may be achieved by transcriptomic regulation (e.g., upregulation of Rho/ROCK axis proteins [145]). In vivo significance of IFNγ-induced permeability seems well-established by the observation that EC-specific IFNGR2-knockout mice showed significantly reduced vascular permeability in an experimental colitis model [146]. Although IFNγ itself belongs to the C1-INH non-sensitive permeability modifying factors, according to multiple studies and our unpublished data, it seems to be the strongest inducer of C1-INH production in ECs and hepatocytes [147–149]. Based on these observations, IFNγ may have a regulatory role in the termination of HAE attacks by elevating C1-INH concentrations.

#### IL-1 Family

Interleukin-1 (IL-1) has two isoforms, IL-1α and IL-1β, both being very potent pro-inflammatory cytokines with pleiotropic effects and key functions in the acute phase reaction, as well as in angiogenesis and permeability regulation. To exert their biological functions, both IL-1 isoforms use the same IL-1 receptor complex (IL-1R1) and both can increase endothelial permeability, although the effect of IL-1β is more pronounced [150]. In vivo and in vitro results suggest that the increased endothelial permeability observed in the IL-1β-treated lung is due to the combination of its direct and indirect effects. IL-1β induces RhoA/ROCK activation, stress fiber formation, and junctional disassembly. Moreover, it activates transforming growth factor β (TGFβ) production in the adjacent epithelial cells, which in turn increases endothelial permeability [151]. IL-1Ra inhibits the permeability-increasing effects of IL-1α and IL-1β as a natural IL-1R antagonist. IL-18 has an ambiguous role in permeability regulation. Although it stimulates the expression of permeability-increasing factors, IL-18 was also shown to have permeability-decreasing and anti-edema effects in the BBB and the retina by upregulating the actin-binding proteins dystrophin and claudin-5, leading to TJ stabilization [152, 153].

#### TGFβ

The anti-inflammatory cytokine Transforming growth factor beta (TGFβ) can also have both permeability-increasing and permeability-decreasing effects. TGFβ, besides its role in angiogenesis, development, and immune-regulation, can mediate endothelial/epithelial barrier protection through a canonical SMAD-dependent stabilization of TJ-associated proteins ZO-1, occludin, and claudin-2 [154, 155], whereas it is also able to induce barrier disruption by non-canonical TGFBR signaling and the activation of the RhoA/ROCK axis [156, 157].

#### IL-10

Interleukin 10 (IL-10), a pleiotropic anti-inflammatory cytokine produced by T_REG_s, B cells, and several other cell types, can decrease EC permeability by inhibiting the production of most pro-inflammatory cytokines. In an in vivo murine model, IL-10 inhibited the increase of microvascular permeability in response to endotoxin [158]. We showed that IL-10 strongly downregulated the expression of VEGF [159], which may also result in the protection of EC barrier function (see below).

#### VEGFs

The most well-characterized interaction of the vascular endothelial growth factor (VEGF) family and their receptors (VEGFR1-3) is between VEGF-A and VEGFR-2. Due to its role in tumor vascularization, it is the target of anti-angiogenic agents. The binding of VEGF-A to EC surface VEGFR-2 induces tyrosine phosphorylation of the receptor and initiates the PLC-γ, PI3K, and p38 MAPK signaling cascades to induce cell proliferation, survival, vasodilation, hyperpermeability, and cell migration [160]. The permeability-increasing effect of VEGF-A—which peaks approximately 15 min after treatment—was found to be dependent mainly on the PI3K-Akt mediated activation of endothelial nitric oxide synthase (eNOS) and nitric oxide (NO) synthesis. Increased NO levels contribute to RhoA activation and cytoskeletal rearrangements, as well as the Src-dependent phosphorylation and destabilization of VE-cadherin in AJs, causing endothelial hyperpermeability [161]. VEGF-A and VEGF-C plasma concentration was found higher in C1-INH-HAE patients in remission than in controls, and the level of both VEGFs further increased during attacks, which supports their major permeability regulating role in BK-mediated angioedemas [162].

#### ANGs

Angiopoietins (ANGs) have a predominant role in the regulation of EC growth, survival, and barrier function. Despite using the same receptor complex (Tie-2 and Tie-1), the effects of ANG-1 and ANG-2 on the vasculature are rather opposite. ANG-1 is produced by mesenchymal cells and is a strong agonist for Tie-2, supporting EC survival, vessel stability, and barrier functions [163]. ANG-1/Tie-2 signaling was shown to further reduce basal permeability and prevent VEGF*-* and thrombin-induced endothelial hyperpermeability by reducing VE-cadherin, occludin, and PECAM-1 phosphorylation, while increasing β-catenin/VE-cadherin and occludin/ZO-1 association, leading to AJ/TJ stabilization [164, 165]. Interestingly, the ANG-1-induced barrier protection against VEGF was found to be mediated by RhoA; however, this did not activate ROCK but another RhoA target mDia, which bound and sequestered Src, depriving VEGFR2 of its essential downstream signaling molecule [166]. Moreover, ANG-1 was shown to be the predominant effector of mesenchymal stem cell (MSC) microvesicles, which have the potential to restore normal barrier function in lung ECs treated with pro-inflammatory cytokines [167].

Recently, ANG-1 has also gained attention among HAE experts as a missense mutation of the *ANGPT1* gene, leading to a reduced ability of the ANG-1 protein to bind Tie-2, was found to be associated with a new type of hereditary angioedema, named ANGPT1-HAE [21]. The underlying mechanism was suggested to be haplo-insufficiency, where the loss-of-function mutant of ANG-1 fails to stabilize VE-cadherin in AJs and could not effectively reduce VEGF- or BK- induced stress fiber formation, leading to increased vascular leakage [168]. ANG-2, on the other hand, can be a weak agonist or an antagonist of Tie-2 in a context-dependent manner. ECs can produce ANG-2 and store it in their Weibel-Palade bodies. Under basal conditions, autocrine ANG-2 may act as a weak Tie-2 agonist in a Tie-1 dependent manner, leading to barrier stabilization. However, during inflammation, ANG-2 expression/release increases, and Tie-1 exodomain cleavage and shedding eliminates Tie-1 from the receptor complex, acting as a molecular switch turning ANG-2 into a Tie-2 antagonist, which causes barrier disruption [163]. In line with the differential permeability modulating effects of ANG-1 and ANG-2, only ANG-2 was found to be elevated during angioedema attacks in C1-INH-HAE patients [162].

#### bFGF

Basic fibroblast growth factor (bFGF) has an important role in angiogenesis and EC proliferation [169]. Recent findings of in vivo experiments imply that BBB protection is achieved by the FGF-receptor mediated activation of the PI3K-Akt-Rac1 signaling pathway that leads to reduced activity of the RhoA/ROCK axis and stabilization of junctional catenins [170]. Additionally, in vitro results suggest the involvement of the ERK signaling in endothelial barrier protection against oxygen–glucose deprivation/reperfusion [171].

#### EGF

Epidermal growth factor (EGF) is an essential growth factor not only for epidermal cells but for almost every epithelial/endothelial/mesothelial cell type. EGF was recently found to protect the BBB after ischemic injury by preventing ischemia-induced decrease of ZO-1 and claudin-5 [172]. Since media of in vitro EC culture usually contain EGF, bFGF and VEGF, it warns us to treat in vitro permeability results with special care, and that the different GF content of these EC media, having opposing effect on EC permeability, may explain several contradictions found in the literature.

### Lipid Mediators

#### PAF

Platelet-activating factor (PAF) is a phospholipid that acts on multiple cell types including ECs. PAF exerts its endothelial effects (i.e., cell migration, angiogenesis, and hyperpermeability) by binding to its G-protein coupled receptor (PAF-R), which signals through Gα_q_ and cSrc, inducing actin cytoskeletal rearrangement and interfering with actin polymerization in a Rac1-dependent but RhoA-independent manner. Very interestingly, PAF-R signaling leads to the disassembly of inter-endothelial junctions without MLC phosphorylation and cell contraction [173].

#### AA Metabolites

The membrane phospholipid derivative arachidonic acid (AA) is the source of many types of eicosanoids important for the regulation of the inflammatory response. These AA metabolites include prostanoids (prostaglandins and thromboxanes), leukotrienes, lipoxins, hydroxyeicosatetraenoic acids (HETEs), and epoxyeicosatrienoic acids. All are potent modulators of vascular function, including endothelial permeability in several cases. Thromboxane A2 (TXA2), as well as prostaglandins PGD2, PGE2, PGF2, and PGI2 exert their strong regulatory function through five basic types of GPCRs, and their effect on vascular permeability depends on the receptor subtype used and the tissue affected [174]. TXA2 for example affects the endothelial barrier acting on the TP prostanoid receptor subtype, activating the Rho/ROCK pathway and disrupting AJs, leading to increased vascular permeability within 15–20 min. This TXA2/TP signaling was found to contribute to tissue edema formation in an acute lung injury model [175], as well as to exacerbate microvascular dysfunction after ischemia/reperfusion [176]. Prostacyclin (PGI2), on the other hand, is a potent barrier protecting prostanoid. It can reduce the LPS- or thrombin-elicited permeability increase by inhibiting RhoA activation through cAMP/Epac or cAMP/Rac1 pathways [177–179].

#### Ceramide and S1P

Ceramide, a sphingolipid, is an important second messenger and mediator of apoptosis in several cell types, including ECs. Ceramide was found to increase endothelial permeability independently of apoptosis and without inducing actin cytoskeleton rearrangement [180]. In contrast to ceramide, its derivative, sphingosine-1-phosphate (S1P), is one of the most potent endothelial barrier protecting agents [181]. Extracellular sphingosine is effectively taken up by red blood cells (RBCs), in which S1P is generated by phosphorylation. RBCs, as the major source of S1P in the plasma, can store S1P in their plasma membranes in large quantities, from where extracellular serum albumin and high-density lipoprotein (HDL) can extract it. HDL and albumin bound S1P is then recognized by its S1P1 receptor (a GPCR) on ECs, and maintains the barrier functions under physiological conditions [182]. Barrier stabilization is achieved via two mechanisms: S1P1 receptor activation leads to a Rac1-dependent stabilization of endothelial junctions [183], and it also inhibits matrix metalloprotease (MMP) activity, thereby protecting EC surface glycocalyx, an important part of the endothelial barrier [184, 185].

### Complement Components

Historically, the complement system was described as the first system involved in BK-mediated angioedema. We previously discussed the role of MASP-1, MASP-2, and C4a that use PARs to regulate endothelial permeability. Here, we focus on two additional molecules of the complement system leaving PAR signaling untouched.

#### C5a

C5a is a potent anaphylatoxin produced by the cleavage of C5 during complement activation. It mediates pro-inflammatory effects via C5aR, a GPCR family member receptor expressed on ECs [186]. In vitro experiments on various types of ECs showed that both C3a and C5a reorganize the actin cytoskeleton, but only C5a is capable of increasing monolayer permeability in a histamine-independent manner [187].

#### C1r

C1r is a serine protease indispensable for the complement classical pathway. We showed that C1r can also increase endothelial permeability, but this effect is independent of its enzymatic activity, therefore, it may not be mediated by PARs [98]. The exact mechanism awaits explanation.

### Purinergic Mediators

#### Adenosine and ATP

Extracellular nucleotides and nucleosides—purinergic mediators—can be derived from every type of cells either by membrane transport or by cellular damage. ECs express numerous types of purinergic receptors, out of which nucleotide signals (e.g., ATP, ADP) are mediated by cation-channel type or GPCR type P2 receptors, while the most important extracellular nucleosides (e.g., adenosine) signal through G-protein-coupled P1 adenosine receptors [188]. Adenosine was shown to enhance the endothelial barrier function in the *vasa vasorum* through stabilization of cortical actin [189] and prevent the detrimental effects of T_H_1 cytokines on the BBB [190], whereas selective activation of the same receptors (A1R and A2AR) was found to be disruptive to the BBB [191]. ATP was also known to either increase or decrease endothelial permeability, but interestingly, newer results suggest that ATP itself induces barrier stabilization through P2Y receptors, while its metabolite adenosine is responsible for the barrier disrupting effect [192].

### Steroid Compounds

#### Estrogens

Besides having an indispensable role in reproduction, estrogens, a group of sex steroids, regulate numerous metabolic and cardiovascular functions. Estrogens decrease the expression of claudin-5 in uterine ECs at the transcription level [193]; however, they restore barrier function in BBB insulted by oxygen–glucose deprivation/reperfusion via classical ERα and ERβ receptors [194]. In most endothelia, estrogens induce HIF1α-dependent VEGF expression [195], which increases permeability as a remote effect. Finally, estrogens were shown to have significant regulatory role in the synthesis of plasma enzyme cascade components relevant to mechanisms of angioedema (i.e., PK and FXII) [22, 196].

#### HC and Dexamethasone

Hydrocortisone (HC) is an anti-inflammatory glucocorticoid frequently used as a supplement to strengthen the barrier function of in vitro cultured ECs. HC decreases the basal vascular permeability by the cAMP signaling pathway as well as diminishes TNFα-induced hyperpermeability in BBB [197]. HC can directly upregulate the expression of occludin in cerebral ECs [198]. Its synthetic derivative, dexamethasone, is frequently used in diseases accompanied by edema, including COVID-19. Besides its anti-inflammatory effects (decreasing pro-inflammatory cytokines), dexamethasone downregulates the expression of VEGF, thus preserving barrier function [199].

#### Danazol

The attenuated androgen danazol has been used as prophylactic agent in C1-INH-HAE for decades. In addition to acting as inducer of C1-INH production in the liver, it has a direct effect on endothelial permeability. In nanomolar doses, it stabilizes the cortical actin ring, thereby protecting barrier function, however, in micromolar concentrations, danazol induces actin stress fiber formation and increases permeability [200]. Its biphasic dose-curve may explain why the effectiveness of danazol has not been straightforward.

### Free Radicals

Free radicals, either generated as byproducts of respiration or as “on purpose” molecules, have numerous biological effects, which occasionally result in completely different outcomes at low and at high concentrations.

#### ROS

Reactive oxygen species (ROS) are produced mainly by different NADPH oxidases (Nox1–5 and Duox1,2) [201]. Superoxide anions (O_2_^−^) generated by Nox1,2,3 and 5 are very potent pro-inflammatory agents via induction of oxidative stress. O_2_^−^ can activate oxidant-sensitive transient receptor potential (TRP) channels that are permeable for Ca^2+^, thereby increased intracellular [Ca^2+^] initiates signaling pathways causing endothelial hyperpermeability [202]. Interestingly, Nox4, Duox1, and Duox2 produce predominantly H_2_O_2_ instead of O_2_^−^, which has (in low concentration) a substantial role in redox signaling and preservation of endothelial barrier function [203].

#### Reactive Nitrogen Species

Nitric oxide (NO) is probably the most pleiotropic effector molecule produced by ECs. Therefore, it is not surprising that NO also has different effects on barrier function, depending on its concentration and the redox state of ECs. When ECs are in a reductive state (i.e., reduced glutathione is present), and produce NO in low nanomolar concentration by eNOS enzyme, RhoA activity is inhibited by its S-nitrosylation, which effectively protects the barrier function [204, 205]. During oxidative stress, however, NO and O_2_^−^ are converted into peroxynitrite (ONOO^−^), which activates RhoA by Tyr-nitration, leading to hyperpermeability [206]. Moreover, NO can mediate the permeability-increasing effects of several factors, including shear stress, thrombin, PAF, ADP, serotonin, VEGF, and BK, when produced in high nanomolar concentration by eNOS [207, 208] or TNFα by inducible NOS (iNOS) [209]. NO may activate cGMP production and MAP kinases, and it can directly bind to catenins entailing their S-nitrosylation and dissociation from VE-cadherin, leading to barrier disruption [208].

### Gasotransmitters and pH

#### H_2_S and CO

Endogenous hydrogen sulfide (H_2_S) and carbon monoxide (CO) are important gasotransmitters in the cardiovascular system with potent anti-inflammatory and barrier protecting effects when produced in a near-physiological concentration [210, 211].

#### Acidic Conditions

Changes in the pH of extracellular fluids may reflect several physiological and pathological processes. ECs highly express proton-sensing receptor GPR4, a GPCR, which elevates permeability in response to acidification through Gα_12/13_/Rho GTPase signaling pathway [212].

### Viral, Fungal, and Bacterial Compounds

Besides endogenous permeability-modifying mediators, microbial factors can also affect the barrier function of the endothelium.

#### Viral Compounds

Viruses predominantly modify the vascular permeability by cytotoxicity, reprogramming the cellular metabolism and inducing cytokine production from the infected cells or from the surrounding leukocytes; however, direct effects were also described. Enterovirus A71 capsid protein VP1 reduced claudin-5 and increased vimentin expression in a murine BBB model, both of which may help the virus to enter the brain [213]. SARS-CoV2 can also modify endothelial permeability either directly or indirectly. The spike glycoprotein of SARS-CoV2 was found to directly induce a rapid decrease in the barrier electrical resistance of endothelial monolayers (with the effect starting immediately and peaking between 12 and 14 h) [214]. The indirect effect of SARS-CoV2 on endothelial permeability involves the depletion of ACE2, a cell surface enzyme serving as a receptor for SARS-CoV2 entry to ECs. As ACE2 is necessary for BK metabolism, its depletion results in the accumulation of des-Arg-BK. It was recently proposed that this BK metabolite binds to BK1R, which is upregulated by pro-inflammatory cytokines in response to the viral activity, thereby enhancing local pulmonary angioedema in a BK-dependent manner [39]. Additionally, many viruses can induce IFNγ production, which may increase C1-INH expression and modulate BK-mediated angioedema.

#### Fungal Compounds

Fungal compounds are also able to modulate vascular permeability. The mycotoxin cytochalasin D induces actin depolymerization leading to junctional destabilization and increased permeability [215].

#### LPS

Bacterial lipopolysaccharide (LPS) is a well-known bacterial permeability-increasing compound, a major component of the Gram-negative outer-membrane. LPS is recognized by a “pattern-recognition receptor,” toll-like receptor 4, expressed by ECs [216, 217]. LPS induces ROCK activation, MLC phosphorylation, stress fiber formation, and disruption of cell junctions, thereby increasing endothelial permeability. Its effect is sustained for up to 24 h and mediated by p38 MAPK, JNK, and NFκB activation, as well as the suppression of barrier stabilizing AMP-activated protein kinase (AMPK) [218, 219]. Moreover, LPS is able to upregulate the receptor expression of several permeability modifying factors in ECs (e.g., BK, histamine, thrombin, and MASP-1) as well as hyperpermeability-inducing cytokines at the site of inflammation (e.g*.*, IL-1β, TNFα) [220–224].

#### Other Bacterial Toxins

*Clostridium difficile* toxin A and B can also strongly disrupt the epithelial barrier independent of cellular damage, mediated by the rearrangement of the actin cytoskeleton and the disintegration of TJs [225], as well as by the upregulation of intestinal VEGF-A production that increases endothelial permeability [226]. *Clostridium botulinum* toxin C2 is also an extremely potent inducer of endothelial hyperpermeability [227] acting via G-actin capping and causing consequent actin depolymerization [228]. Pertussis toxin from *Bordetella pertussis* also increases permeability in low concentrations without modifying actin cytoskeleton or junctional molecules; however, in higher concentrations, it protects barrier function by stimulating cAMP production [229]. Cholera toxin from *Vibrio cholerae*, on the other hand, is known to decrease endothelial permeability by the stimulation of cAMP production, which leads to the inhibition of MLC phosphorylation and acto-myosin contraction and thereby enhances the endothelial barrier and counteracts the effects of barrier destabilizing agents [230, 231]. Another toxin produced by *Vibrio cholerae* is zonula occludens toxin (Zot), which mimics the effects of an endogenous protein, zonulin (pre-haptoglobin 2, a serine-protease homolog). Both Zot and zonulin contain a PAR2 activating motif and increase permeability through PAR2 activated, PKCα-dependent phosphorylation and displacement of TJ component ZO-1, as well as cytoskeletal rearrangement [232, 233]. Although Zot and zonulin were thought to be involved in the regulation of gastrointestinal tract epithelial cell permeability, similar effects were shown in a lung model, involving both epithelial cells and ECs [234].

Although there is no compelling evidence whether microbial compounds indeed directly trigger angioedema attacks, we previously described a decreased frequency of abdominal attacks in C1-INH-HAE patients following eradication of the stomach mucosal-resident pathogen *Helicobacter pylori* [235], which suggests that bacterial macromolecules may indeed be permeability modifying trigger-factors either directly, or via the activation of immune and/or neural system.

## Current Controversies in BK-Mediated Angioedema

During the more than 130-year-long period that has elapsed since hereditary angioedema was first described, many details of its pathomechanism have been elucidated. However, the “pieces of the puzzle” have not yet come together. Many questions and contradictions remain unanswered, and there are phenomena that appear controversial due to the lack of sufficient information.

### The Mystery of Angioedema Localization

Why do angioedema attacks manifest only in certain body regions, whereas others are spared? Upper airway edema involves the mucosa of the mesopharynx, the larynx and the hypopharynx. Why does it spare the nasal or the bronchial mucosa? Why are the skin and the gastrointestinal tract frequently affected, but others like the lungs, heart, liver or kidneys are not? These questions puzzle many experts in the angioedema field and we possess some plausible answers. Hofman et al. speculated how a systemic activation due to lack of inhibitory effect of C1-INH leads to local manifestations [236]. We would like to add some novel hints to complement the aforementioned explanation.

**Fig. 3:**
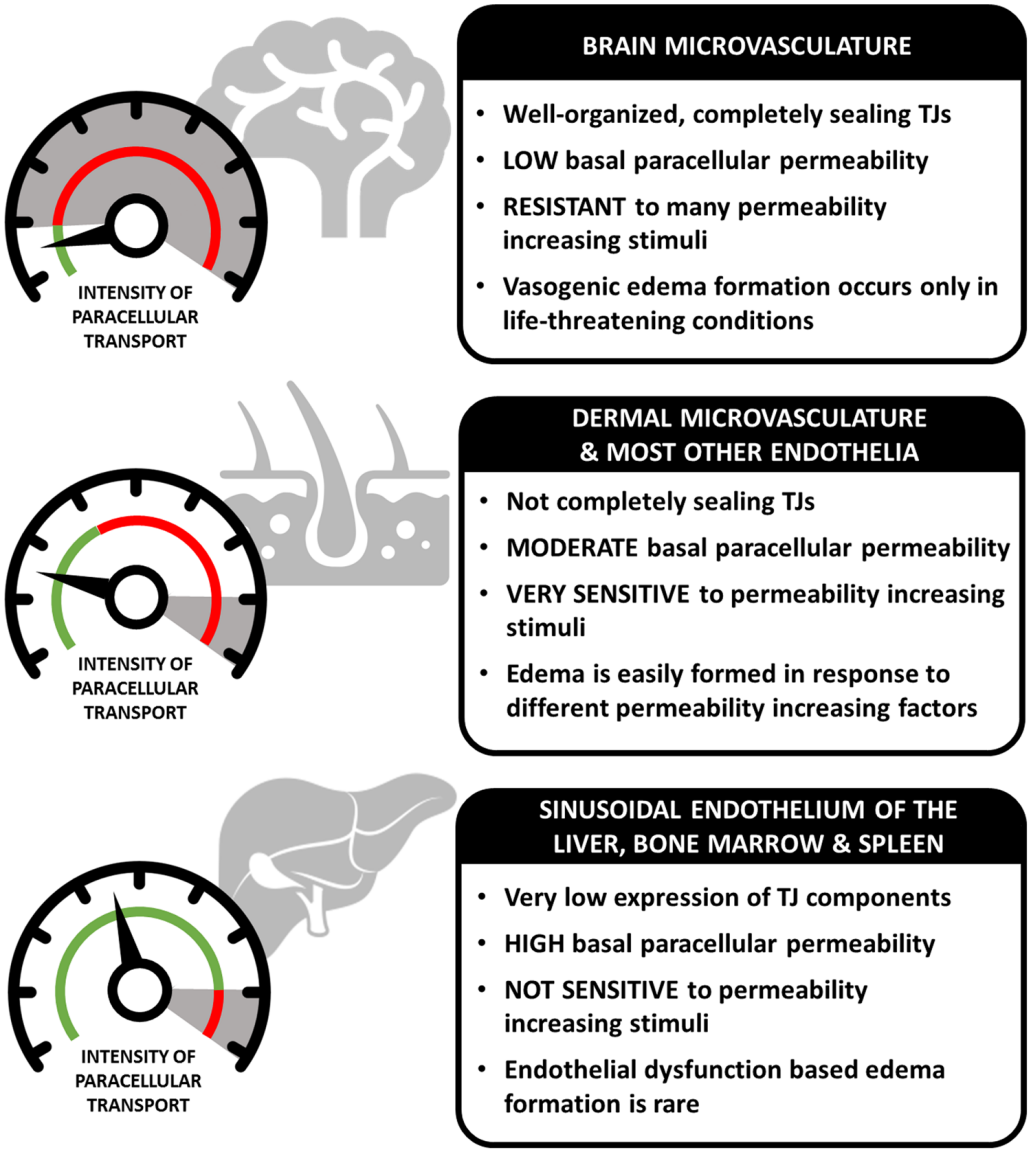
Characteristics of endothelial permeability regulation in different tissues. The brain, skin and liver are shown as examples. The green area indicates normal activity, whereas the red area indicates pathological intensity of paracellular transport. Gray areas on the dials indicate zone of permeability that is rarely reached even in pathological conditions (never or very few times during a lifetime). Note that skin microvasculature becomes frequently hyperpermeable (indicated by red line without grey area) during a normal lifespan (e.g., in response to minor traumas, mosquito bites, allergic reactions, etc.)

ECs show significant functional and morphological heterogeneity [237], which causes differences in basal and inducible permeability state across the vascular system (Fig. 3). As an example, expression pattern of junctional molecules claudin-5, VE-cadherin, occludin, ZO-1, and JAM-C is different in human dermal microvascular EC (HDMEC), lung MEC, HUVEC, and aortic EC [238]. Although the resting pattern of leukocyte homing receptors (ICAM-1, VCAM-1, and E-selectin) is similar in HUVECs and human intestinal MECs, the kinetics of induction as well as inducibility by pro-inflammatory factors are fairly different [239]. Another example is the case of four histamine receptors, which are present on HUVECs, HDMECs, and human cerebral MECs, but their distribution is distinct, and induced trans-endothelial resistance showed different kinetics and recovery time [125]. It appears that although PAR1,2 and 4 are widely expressed in the human body, their distribution in ECs from various anatomical sites differs significantly. PAR1 is usually the most abundant, followed by PAR2 then PAR4, however, the pattern of their relative expression is different amongst pulmonary, dermal, and umbilical cord ECs [240–244]. Also, iNOS inhibitors increase binding of leukocytes to cytokine- and LPS-activated human intestinal MECs but not to similarly activated HUVECs [245]. The differences in induction of permeability are also observed at the functional level. Basal albumin leak was found to be significantly lower in human pulmonary MECs (HPMEC) than in HUVECs, but septicemia increases the leak across both types. Moreover, the presence of neutrophils enhances sepsis-increased albumin leak in HPMEC but not in HUVECs [246].

Taken together, the very same permeability-increasing stimuli may provoke significantly different response in various parts of the vascular tree, which adds an “EC perspective” to the enigma of edema localization in angioedema.

### The Secret of Unpredictable Occurrence and Severity of Angioedema Attacks

Why do HAE patients not experience continuous attacks, while their C1-INH level is permanently low? Why there is no correlation between C1-INH level and severity of the disease? Why does mechanical trauma (as a triggering factor) provoke an attack on one occasion but not on another? These questions may indicate that BK-mediated angioedema comprises a heterogeneous group of multifactorial diseases, even if their pathomechanism can be linked to mutations of a single gene (*SERPING1*, *FXII*, *ANGPT1*, etc*.*) or to an extrinsic factor (e.g., ACE- and DPP-IV-inhibitors).

**Fig. 4:**
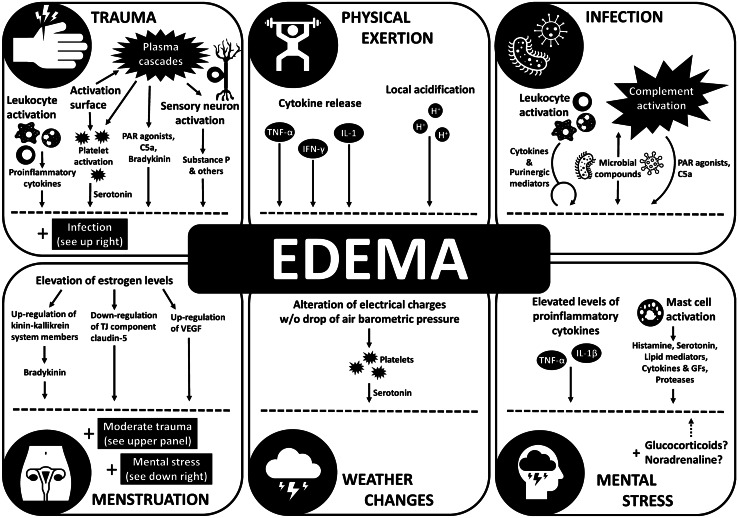
Proposed mechanisms of common trigger-factors of angioedema attacks. The figure shows the most important mechanisms by which trigger factors of hereditary angioedema exert their vascular permeability effects on endothelial cells

Trigger-factors, acting either alone or in combination, are also able to modify vascular permeability. Therefore, together with the preexisting genetic and environmental background, they contribute to defining the frequency, severity, and localization of the attacks. Figure 4 summarizes the permeability-increasing effects of trigger-factors identified or suspected in C1-INH-HAE [247].

Even minor *trauma* has multiple effects on permeability. Activation of coagulation and fibrinolytic pathways forms thrombin and other PAR agonist enzymes from their zymogens, which have direct effects on ECs. Negatively charged surfaces activate the KKS and generate BK, but activated enzymes of KKS concurrently act directly on ECs (via PARs) and stimulate sensory neurons, which, in turn, enhance permeability by the secretion of SP and other neurotransmitters [119]. Finally, even “sterile” trauma may induce local inflammation, which involves the complement system and leukocytes, thereby increasing permeability through MASP-1, anaphylatoxins, and pro-inflammatory cytokines. Pathomechanism of non-sterile trauma also involves microbial compounds with permeability-enhancing potentials.

*Physical exertion* was also shown to induce pro-inflammatory cytokines including those that increase permeability (IL-1, TNFα, IFNγ) [248], and to cause micro-injuries to the skeletal muscles (sterile trauma). Moreover, local acidification of the muscles by accumulated lactic acid increases permeability through the pH-sensing GPR4 [212].

*Menstruation* is one of the most frequent and reliably identified trigger-factors of angioedema attacks in females. However, its effect on permeability is very complex. Besides being a sort of moderate trauma, steroid hormone pattern changes dramatically during menstruation. The first peak of estrogen precedes the beginning of menstruation by 1–2 days, which coincides with the long-onset down-regulation of claudin-5 in endometrial ECs [193]. VEGF, PK, and FXII inducing capability of estrogens may explain the remote permeability-increasing effects. Finally, menstruation may evidently cause mental stress in a great proportion of individuals (see below).

*Pregnancy* may alleviate or aggravate the angioedema attacks. Interestingly, C1-INH-HAE patients being pregnant with fetus afflicted by C1-INH-HAE register significantly more attacks than those with healthy fetus [249]. Since C1-INH is supposed to be unable to cross fetal-maternal interface, other, not yet identified trigger-factors should disturb the maternal P/B state in these cases.

*Weather changes* comprise a drift in air-pressure, temperature, and/or atmospheric electrical charges. Although their exact mechanism on human physiology is still the objects of extended studies, alteration in electrical charges is long known to interfere with the circadian-rhythm by the release of serotonin [250], or by the changes of serotonin responsiveness [251]. Since serotonin can modulate permeability [252], weather changes may directly trigger angioedema attacks by serotonin release.

Impact of *mental stress* on angioedema formation is still largely unexplored. Even if solid evidence is missing, there are some putative explanations. Mental stress causes hemoconcentration in otherwise healthy people, which entail subclinical systemic increase in endothelial permeability. In patients with angioedema, at some predisposed locations, together with elevated BK level or decreased activity of ANG-1, mental stress may trigger angioedema attacks. Although noradrenaline was shown to be elevated in a tilt-induced stress model of C1-INH-HAE patients [23], the pathogenetic role of noradrenaline in increasing permeability is controversial [132–134]. Glucocorticoids are also increased during stress; however, their effects are questionable similarly to catecholamines, as glucocorticoids decrease permeability directly [197] as well as indirectly by reducing pro-inflammatory cytokine production of T cells and other leukocytes. Nevertheless, IL-1β and TNFα levels are significantly elevated during stress [253], which may contribute to the increased permeability, but the exact mechanism leading to the elevation of these pro-inflammatory cytokines in the presence of the high cortisol conditions is still not clear. A more promising explanation is stress-induced MC activation [254], in which numerous compounds that increase endothelial permeability are released, including histamine, serotonin, lipid mediators, cytokines, growth factors, and proteases [255].

*Infection* can induce angioedema by several related mechanisms. Microbial compounds themselves may trigger the loss of barrier function, and they also modulate permeability via activating the complement cascade, leukocytes, and cells of the infected tissue locally, which results the release of pro-inflammatory cytokines, purinergic mediators, and PAR agonist enzymes.

Certain *drugs* hindering BK catabolism, such as ACE inhibitors and DPP-IV inhibitors, can cause angioedema that is usually regarded as an acquired form. These drugs may also be potential trigger-factors in HAE.

### The Riddle of Response to Therapy

Why does the administration of C1-INH concentrate prove effective in BK-mediated angioedema despite normal plasma levels of C1-INH? In contrast, why do patients with BK-mediated angioedema have any remaining active C1-INH before or during angioedema attacks? Why is the concentration of C1-INH complexes with plasma enzymes similar in healthy people and in patients with angioedema [256, 257]?

C1-INH concentrate can effectively improve or terminate attacks even in patients with normal C1-INH levels, such as in ACEI-AAE patients [38], which underlines the permeability signal integrating role of ECs. It can be hypothesized that in patients with BK-mediated angioedema, excess of active serine proteases cause a low and constant, but still controllable permeability stimulation to ECs; however, edema formation requires an additional trigger-factor. We and others demonstrated that circulating C1-INH is complexed with several plasma serine proteases both in healthy individuals and C1-INH-HAE patients [256, 257], which supports the hypothesis that a small fraction of these enzymes is activated spontaneously and continuously (tick-over mechanism). Administration of C1-INH inhibits serine proteases, lowers the basal permeability, and thus prevents the effect of the trigger-factor to reach the threshold for edema formation.

Another paradox is that C1-INH is not completely depleted during attacks. This can be resolved by the fact that angioedema does not depend on a single serine protease. The extra permeability-increasing effect of the trigger-factors, with or without minor elevation in PKa activity [256] is sufficient to initiate an attack when ECs are pre-sensitized by BK, PKa, or other serine proteases. The P/B state of ECs relies on a fine-tuned delicate balance. The above observations indicate that the P/B state can be impaired not only by a drastic alteration in one particular compound, but by tiny changes in many factors impacting the ECs.

## Conclusions and Future Perspectives

ECs have a great variety of physiological functions. At the level of microcirculation, one of their most important functions is the regulation of a proper P/B state. ECs act as signal integrators with a vast repertoire of cell surface receptors, adhesion molecules and a highly developed cytoskeleton system, by which they respond to the requirements of their microenvironment. ECs also have several built-in control mechanisms to shut down exaggerated modifications of the P/B state. One of the most well-studied negative feed-back mechanisms is receptor desensitization, achieved either by irreversible impairment of the receptor (PARs) or by endocytosis (e.g., BK receptors, histamine receptors). Thus, in the majority of cases, including angioedema, multiple impacts on ECs are needed to form a sustained hyperpermeability state leading to edema. This would explain the great number of controversial findings about BK-mediated angioedemas described in the previous chapter.

**Fig. 5:**
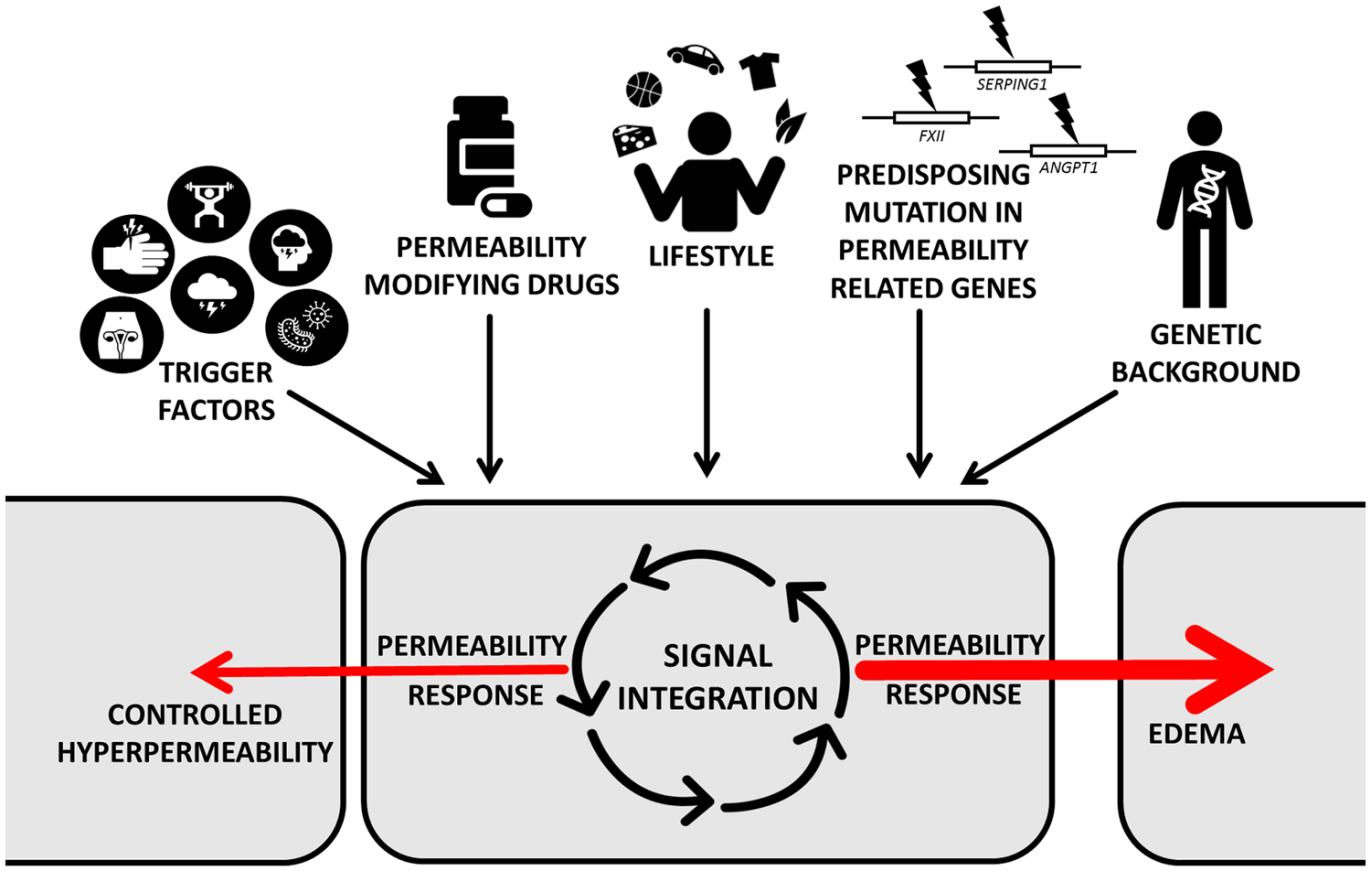
Role of endothelial signal integration in the permeability response. Predisposing mutations and genetic background together with ever-changing factors (i.e., drugs, lifestyle, and trigger-factors) act on endothelial cells, which integrate these signals. The output of this signal integration (i.e., controlled hyperpermeability or uncontrolled edema) could only be estimated if each of these signals were fully known

Taken together, we attempt to outline how angioedema attacks evolve (Fig. 5). First, the genetic background determines how susceptible the ECs are to different permeability inducing agents in general. This is modulated by the person’s lifestyle and other environmental effects, which may explain why there is such big a difference in attack frequency, severity, and localization even in the same type of angioedema. Secondly, a strong permeability increasing impact is required, which elevates either the permeability or the inducibility of ECs in response to permeability increasing agents. A mutation in any of the permeability-related genes (e.g., *SERPING1*,* FXII*,* ANGPT1*) or a permeability modifying drug (i.e., ACE or DPP-IV inhibitors) can contribute to such instability. Such an effect may differ amongst organs because of their diverse endothelial lining. The third component is the actual trigger-factor that pushes the permeability over the limit of controllable range.

The edema resolves without medical intervention, when (1) the sum concentration of the permeability increasing factors drops below a certain level, (2) the sum concentration of the barrier supporting factors elevates as a built-in barrier protecting mechanism, and (3) the receptors of permeability increasing factors are desensitized at protein or transcriptional level, or because of the natural combination of the previous mechanisms.

To understand the complex pathomechanism of angioedema, research strategy should be directed toward simultaneous investigation of several stimuli that act on ECs. Also, utilization of genome-, transcriptome-, proteome-, and metabolome-based systems-biological approaches would enable to study the simultaneous, multiple effects on ECs. International collaborations resulting in high-number, standardized, good quality samples (bio-banking supported with fully registered clinical data) would allow us to recognize weak-interactions and network effects. Improved analytics would be also required to measure crucial factors (e.g., BK itself) in a quantitative, reproducible, and affordable manner. Interstitial fluid samples taken from the very site of edema would help comprehend the local driving forces of endothelial based, BK-mediated angioedema. These methodological advancements in angioedema research together will be necessary to understand and reverse the deleterious effects of permeability-increasing factors, the molecular dambusters.

## Abbreviations

AA: Arachidonic acid
ACE: Angiotensin-converting enzyme
ACEI: Angiotensin-converting enzyme inhibitor
ADM: Adrenomedullin
AJ: Adherent junction
AMPK: AMP activated protein kinase
ANGs: Angiopoietins
AVP: Arginine-vasopressin
BAEC: Bovine aortic endothelial cell
BBB: Blood–brain barrier
bFGF: Basic fibroblast growth factor
BK: Bradykinin
BKRB1: Bradykinin receptor B1
BKRB2: Bradykinin receptor B2
C1-INH: C1-inhibitor
cAMP: Cyclic adenosine monophosphate
CGRP: Calcitonin gene-related-peptide
CLS: Capillary leak syndrome
CNS: Central nervous system
CO: Carbon monoxide
DPP-IV: Dipeptidyl peptidase 4
DS: Desmosome
ECs: Endothelial cells
EGF: Epidermal growth factor
eNOS: Endothelial nitric oxide synthase
ET-1: Endothelin-1
GF: Growth factor
GPCR: G-protein coupled receptor
H_2_O_2_: Hydrogen peroxide
HAE: Hereditary angioedema
HAEC: Human aortic endothelial cell
HC: Hydrocortisone
HD: Hemidesmosome
HDL: High-density lipoprotein
HDMEC: Human dermal microvascular endothelial cell
HETE: Hydroxyeicosatetraenoic acid
HK: High molecular weight kininogen
HPMEC: Human pulmonary microvascular endothelial cell
H/R: Hypoxia/reoxygenation
HUVEC: Human umbilical vein endothelial cell
HVP: High vascular permeability
IFNγ: Interferon γ
iNOS: Inducible nitric oxide synthase
IL: Interleukin
IVP: Increased vascular permeability
JAM: Junctional adhesion molecule
KKS: Kallikrein kinin system
KLK-1: Tissue kallikrein 1
LPS: Lipopolysaccharide
MASP-1: Mannan-binding lectin-associated serine protease 1
MASP-2: Mannan-binding lectin-associated serine protease 2
MC: Mast cell
MLC: Myosin light chain
MLCK: Myosin light chain kinase
MMP: Matrix metalloprotease
MSC: Mesenchymal stem cell
NK: Neurokinin
NO: Nitric oxide
NP: Natriuretic peptide
PACAP: Pituitary adenylate cyclase-activating polypeptide
PAR: Protease activated receptor
P/B state: Permeability/barrier state
PAF: Platelet-activating factor
PGI2: Prostacyclin
PK: Prekallikrein
PKa: Plasma kallikrein
PKB: Protein kinase B
PKC: Protein kinase C
PPD: Paroxysmal permeability disorder
RBC: Red blood cell
ROS: Reactive oxygen species
SP: Substance P
S1P: Sphingosine-1-phosphate
TGFβ: Transforming growth factor β
TJ: Tight junction
TNFα: Tumor necrosis factor α
TRP: Transient receptor potential
TXA2: Thromboxane A2
VEGF: Vascular growth factor
VIP: Vasoactive intestinal peptide
ZO: Zonula occludens
Zot: Zonula occludens toxin
5-HT: Serotonin/5-hydroxytryptamine
